# WITHIN-PERSON ASSOCIATIONS BETWEEN MARITAL DISSOLUTION AND SUBSEQUENT COGNITIVE FUNCTION IN MIDDLE AND LATER LIFE

**DOI:** 10.1093/geroni/igad104.2140

**Published:** 2023-12-21

**Authors:** Julia Tucker, Sae Hwang Han

**Affiliations:** University of Texas at Austin, Austin, Texas, United States; University of Texas at Austin, Austin, Texas, United States

## Abstract

In light of the growing burden associated with cognitive impairment and dementias on families and society accompanied by the dramatic shifts in late-life family demography, there is growing literature focusing on the relationship between marital biography and cognitive outcomes in middle and late adulthood. This study aimed to contribute to the literature by examining within-person linkages between cognitive outcomes associated with two forms of marital dissolutions experienced in later life—divorce and widowhood. Framed around the key concepts of the life course perspective, we examined within-person changes in cognitive function associated with divorce and widowhood transitions in middle and late adulthood. Drawing from 11 waves of longitudinal data from the Health and Retirement Study (1998-2018), we followed over twenty-three thousand married individuals 50 and older over the study period that spanned nearly two decades (person-wave observations N=128,033). Cognitive function at each wave was evaluated using a modified version of the Telephone Interview for Cognitive Status (m-TICS). Research aims were addressed using within-between random effects models that yielded within-person estimates unconfounded by person-level characteristics. Approximately 4.4% and 17.7% of the study sample experienced divorce and widowhood transitions during the observation period. The model results indicated that divorce transitions were unrelated to changes in cognitive function. Cognitive outcomes associated with widowhood transitions were heterogeneous for women and men, where women who transitioned to widowhood experienced increases in cognitive function, whereas men experienced reductions in cognitive function. These findings are discussed in the context of implications for practice and policy.

